# Comparison between observed children's tooth brushing habits and those reported by mothers

**DOI:** 10.1186/1472-6831-11-22

**Published:** 2011-09-03

**Authors:** Carolina C Martins, Maria J Oliveira, Isabela A Pordeus, Saul M Paiva

**Affiliations:** 1 Department Pediatric Dentistry and Orthodontics, School of Dentistry, Federal University of Minas Gerais, Belo Horizonte, Brazil; 2 Department Pediatric Dentistry and Orthodontics, School of Dentistry, State University of Montes Claros, Montes Claros, Brazil

## Abstract

**Background:**

Information bias can occur in epidemiological studies and compromise scientific outcomes, especially when evaluating information given by a patient regarding their own health. The oral habits of children reported by their mothers are commonly used to evaluate tooth brushing practices and to estimate fluoride intake by children. The aim of the present study was to compare observed tooth-brushing habits of young children using fluoridated toothpaste with those reported by mothers.

**Methods:**

A sample of 201 mothers and their children (aged 24-48 months) from Montes Claros, Brazil, took part in a cross-sectional study. At day-care centres, the mothers answered a self-administered questionnaire on their child's tooth-brushing habits. The structured questionnaire had six items with two to three possible answers. An appointment was then made with each mother/child pair at day-care centres. The participants were asked to demonstrate the tooth-brushing practice as usually performed at home. A trained examiner observed and documented the procedure. Observed tooth brushing and that reported by mothers were compared for overall agreement using Cohen's Kappa coefficient and the McNemar test.

**Results:**

Cohen's Kappa values comparing mothers' reports and tooth brushing observed by the examiner ranged from poor-to-good (0.00-0.75). There were statistically significant differences between observed tooth brushing habits and those reported by mothers (p < 0.001). When observed by the examiner, the frequencies of dentifrice dispersed on all bristles (35.9%), children who brushed their teeth alone (33.8%) and those who did not rinse their mouths during brushing (42.0%) were higher than those reported by the mothers (12.1%, 18.9% and 6.5%, respectively; p < 0.001).

**Conclusions:**

In general, there was low agreement between observed tooth brushing and mothers' reports. Moreover, the different methods of estimation resulted in differences in the frequencies of tooth brushing habits, indicative of reporting bias. Data regarding children's tooth-brushing habits as reported by mothers should be considered with caution in epidemiological surveys on fluoridated dentifrice use and the risk of dental fluorosis.

## Background

Information bias can occur in epidemiological studies and compromise the scientific outcome, especially when evaluating patient self-report regarding their own health. The aim of the study design is to obtain the most accurate results to represent reality. In paediatric dentistry, a number of studies have evaluated fluoride intake among children either from dietary sources or from tooth-brushing with fluoridated dentifrice [1-4]. The current method used to evaluate fluoride intake from diet is the duplicate plate method [1]. A comparison between the duplicate plate method and dietary logs provided by parents' suggest differences in fluoride intake by children between methods, with the dietary log reporting significantly higher fluoride intake from food than the duplicate plate method [5]. However, the few other studies comparing methodologies for food and beverage consumption on the part of young children focus on the risk of obesity [6,7].

To estimate the risk of dental fluorosis in young children, fluoride intake also considers tooth brushing with fluoridated dentifrices. Young children often ingest a large proportion of the dentifrice dispersed on the toothbrush, thereby increasing the risk of developing dental fluorosis [3,4]. One method for evaluating children's tooth brushing habits is through direct observation. This method was used as the gold standard for comparison with another method in a previous study [8]. The use of mothers' reports regarding their children's tooth-brushing habits is another commonly employed method. One study found statistically significant differences in mothers' reports regarding their children's brushing habits when the same interview was repeated six years later, suggesting recall bias [9]. However, there is a lack of studies determining whether mothers' reports are similar to the actual brushing habits of their children. As reliable data are important for the assessment of risk factors, information bias can compromise the results. For example, the daily frequency of tooth-brushing is used to calculate a child's daily fluoride intake from dentifrice [3]. Thus, the over-reporting of daily tooth-brushing frequency on the part of mothers leads to an over-estimation of children's daily fluoride intake. Considering the lack of studies comparing information collected using different methodologies, it is important to evaluate these data to determine the most reliable and valid method.

The aim of the present study was to compare the agreement between observed children's tooth-brushing habits using fluoridated toothpastes with the habits reported by mothers.

## Methods

The present study was conducted as part of a larger cross-sectional study on fluoride intake by dentifrices among children from Montes Claros, MG, Brazil. Eight day-care centres in the city of Montes Claros (four public and four private) were randomly selected from a list of day-care centres compiled by the Municipal Department of Education. At the time of data collection (2007-2008), Montes Claros had 84 day-care centres (31 public and 53 private), at which 3,898 children were enrolled [10]. The directors of these centres were contacted and consented to the conduction of the study. A meeting was initially set up with parents, at which time the mothers received information about the objectives and signed terms of informed consent authorising their participation in the study. It was made clear that the presence of mothers was preferred over fathers and, thus, only mothers were present at the meeting. The following were the inclusion criteria to take part in the study: mothers must be present at the meeting and their children must be between 24-48 months of age. Five mothers with children less than 24 months of age were excluded from the study. The initial sample comprised 203 mothers among whom two failed to complete the questionnaire and were excluded from the study. The final sample comprised 201 pairs of mothers and children (0.98% of drop-outs). The mean age of the children was 41.3 months. The total number of mothers and children attending the day-care centres was 362. All mothers that were a) present at the meeting and b) agreed to participate were included in the study.

Data collection was carried out from October 2007-June 2008. The study received approval from the Human Research Ethics Committee of the Federal University of Minas Gerais (ETIC 278/07).

### Mothers' reports

Mothers who agreed to participate by signing an informed consent form were then asked to answer a structured questionnaire on their children's current tooth-brushing habits. A self-administered questionnaire was distributed by one of the examiners (MJO) to be filled out by the mothers at the day-care centres who were instructed that there were no right or wrong answers and that they should answer the questionnaire based on their children's tooth-brushing habits at home. The questionnaire was structurally composed of six items-five with two options and one with three options (Table 1). The first item ("kind of dentifrice the child uses") had two options: "children's" and "adults'". In Brazil, children's dentifrices are specially flavoured for children (fruit, gum, strawberry, grape, etc.) and usually contain between 0-1100 ppm F. Adults' dentifrices are mint flavoured and contain between 1100-1500 ppm F [11]. Brand names were maintained in the questionnaire to enhance respondent comprehension.

**Table 1:** Comparison of mothers' reports and tooth brushing habits observed by examiner

		Observed by the examiner							
	**Kind of dentifrice child uses**		**Children's**	**Adult's**		**Prevalence** **%**	**p-value†**	**Agreement %**	**K** (SE*)
		**Children's**	92	7		49.3	0.043	87.6	0.75 (0.05)
		**Adult's**	18	84		50.7			
		**Prevalence %**	54.7	45.3					
	
	**Amount of dentifrice**		**- 1/2 of bristles**	**1/2 of bristles**	**All bristles**	**Prevalence** **%**	**p-value†**	**Agreement %**	**K** (SE*)
	**dispensed on brush**	**- 1/2 of bristles**	47	28	8	41.9	< 0.001	47.0	0.22 (0.05)
		**1/2 of bristles**	18	28	45	46.0			
		**All bristles**	2	4	18	12.1			
		**Prevalence %**	33.8	30.3	35.9				
	
**Mothers' report**	**Who dispenses**		**Child alone**	**An adult**		**Prevalence** **%**	**p-value†**	**Agreement %**	**K** (SE*)
	**dentifrice on toothbrush?**	**Child alone**	0	38		18.9	< 0.001	80.6	0.00 (0.01)
		**An adult**	1	162		81.1			
		**Prevalence %**	0.5	99.5					
	
	**Who brushes child's teeth?**		**Child alone**	**An adult**		**Prevalence** **%**	**p-value†**	**Agreement %**	**K** (SE*)
		**Child alone**	30	8		18.9	< 0.001	77.1	0.43 (0.07)
		**An adult**	39	125		81.1			
		**Prevalence %**	33.8	66.2					
	
	**Does child spit out**		**Yes**	**No**		**Prevalence** **%**	**p-value†**	**Agreement %**	**K** (SE*)
	**dentifrice during**	**Yes**	67	11		39.6	< 0.001	50.2	0.11 (0.05)
	**brushing?**	**No**	87	32		60.4			
		**Prevalence %**	78.2	21.8					
	
	**Does child rinse out**		**No**	**Yes**		**Prevalence** **%**	**p-value†**	**Agreement %**	**K** (SE*)
	**mouth during brushing?**	**No**	10	3		6.5	< 0.001	61.5	0.11 (0.04)
		**Yes**	74	113		93.5			
			**Prevalence %**	42.0	58.0				

*Standard error; ^† ^McNemar test

### Tooth-brushing observed by an examiner

In the second part of the study, an appointment was made with the mothers and their children at the day-care centre one week after answering the questionnaire. Mothers were asked to bring the dentifrice and toothbrush that the child used at home. An appointment was made for each mother/child pair separately, without the presence of other mothers. The mothers and children were led to a bathroom and asked to perform tooth-brushing, reproducing the same technique normally employed at home. No instructions on tooth-brushing were given. An examiner (MJO) observed without intervening and recorded notes on a structured form, which contained the same items and response options as the questionnaire (Table 1). The examiner maintained sufficient distance while observing to avoid disturbing the normal routine of the process. The tooth-brushing practice of each mother/child pair was observed only once.

The examiner was an experienced paediatric dentist who underwent a training process for the observation of tooth-brushing. The calibration process is described below.

### Calibration process

Before the main study, a training process was conducted to ensure that the participants would understand the method. For this, a day-care centre that was not part of the main sample was chosen. Ten mother/child pairs took part in this process. The mothers were asked to answer the questionnaire and all items were fully understood. Each mother/child pair was then asked to perform tooth-brushing at the day-care centre. There were no major corrections required for the calibration process and the main study was then conducted. In this part of the study, the examiner was able to improve the observation method.

### Statistical analysis

Data were analyzed using the Statistical Package for Social Sciences (SPSS for Windows, version 12.0, SPSS Inc., Chicago, IL, USA). Mothers' reports were compared with observed tooth-brushing and the data were analysed for overall agreement (%) and Cohen's Kappa coefficient. To calculate the Kappa coefficient, the following formula was used: $k=\frac{po-pe}{1-pe}$, in which 'po' is the proportion of units with agreement: $po=\frac{a+d}{a+b+c+d}$ and 'pe' is the proportion of units for which agreement is expected by chance: $\frac{\left[\left(a+b\right).\left(a+c\right)\right]+\left[\left(c+d\right).\left(b+d\right)\right]}{{\left(a+b+c+d\right)}^{2}}$[12]. Agreement strength was based on the following criteria: 0.00-0.20 = 'poor'; 0.21-0.40 = 'fair'; 0.41-0.60 = 'moderate'; 0.61-0.80 = 'good'; 0.81-1.00 = 'very good' [13]. Overall agreement considers the proportion of total agreement divided by the total (po) and not by chance (Kappa). The McNemar test was used to compare the reported frequency of tooth-brushing habits with that observed by the examiner (level of significance set at 5%). Missing data were "I don't know" answers and those left blank on the questionnaire, which were not considered in the analyses.

## Results

Eighty-nine children were male (44.3%) and 112 were female (55.7%); 72 children were from private day-care centres (35.8%) and 129 were from public day-care centres (64.2%). Table 1 displays the comparison between the observed data and those from the mothers' reports.

Kappa agreement ranged from 0.00-0.75. The "kind of dentifrice the child uses" achieved the best agreement (good, K = 0.75, standard error = 0.05) and the highest overall agreement (87.6%). All other items achieved moderate to poor agreement (K = 0.43-0.00). Overall agreement ranged from 47.0-87.6%. Table 1 shows agreement values, where a particular reported behaviour was confirmed by observation. For example, 10 mothers reported that their child did not rinse his/her mouth during tooth-brushing, which was also observed by the examiner (last question). Mouth-rinsing by children during brushing was reported by 113 mothers, which was in agreement with the observed finding. For the remaining 77 answers, the observed data did not match those reported by the mothers.

Comparisons of frequencies between the observed results and mothers' reports were statistically different on all questions (McNemar test, p < 0.05) (Table 1). The frequencies of children's dentifrice use (54.7%), amount of dentifrice dispersed on all bristles (35.9%) and the number of children that spit out the dentifrice (78.2%) were significantly higher when observed by the examiner than when reported by mothers (49.3%, 12.1% and 39.6%, respectively; p < 0.05, McNemar test). In contrast, the frequencies of adults who brushed the child's teeth (81.1%) and children who rinsed their mouth out during brushing (93.5%) were significantly higher when reported by mothers than observed by the examiner (66.2% and 58.0%, respectively; p < 0.001).

## Discussion

The most widely employed methods for evaluating patient-reported health conditions are interviews and questionnaires [14-17]. However, information from patients can be biased because of forgotten past episodes or to over-reporting of certain habits to appear careful about one's health.

In the present study, the majority of children were enrolled in public day-care centres (64.2%) and the remainder were enrolled in private day-care centres (35.8%). These institutions mainly carried out teaching activities directed at preschool children. No institution offered a class in oral health. The education was similar in both groups of children. Thus, the oral health habits of these children were not influenced by the learning process at school.

Parental opinion is considered a valuable tool for the assessment of children's conditions. A previous Brazilian study tested the validity of mothers' opinions regarding their child's life [18]. Our decision to collect data at day care centres was made because many mothers leave their children at such centres while working, rather than leaving them with nannies or grandparents. Moreover, there is great diversity in the types of mothers at day-care centres. Brazil is a predominantly catholic country with married, divorced and single mothers. Many married women work to help with family finances, at salaries that can be lower or higher than that of their husband. There are also married mothers who do not work and commit themselves entirely to family care. In divorce, Brazilian law gives the woman priority regarding the guardianship of a child and, finally, there is a proportion of single mothers who live with their parents or who are the head of the family. In the city of Montes Claros, 81% of preschool children were not enrolled at day-care centres [19].

A previous study carried out in Brazil demonstrated mothers' comprehension of their children's cognitive, psychological, emotional and physical development. The study also found that mothers consider their presence of great importance in raising and educating their children, more so than the father, who is considered important mainly as a male role model [20].

Kappa values were mainly low, ranging from poor to moderate. Overall agreement was higher than the Kappa value in most cases, which corroborates the findings of previous studies [9,21]. This is because the Kappa index applies adjusted measures for random agreement when the same fact is evaluated twice [12]. The item "kind of dentifrice that the child uses" achieved the highest overall agreement and good Kappa agreement. This was perhaps the easiest question and it is likely that the mothers were responsible for buying the dentifrice, making it easy to remember. Other questions contained details that were harder to remember, such as whether the child spat the paste out or the amount of dentifrice used. Another factor that could have been related to the poor Kappa values on these two items is the fact that young children are still developing their tooth-brushing habits, so spitting and rinsing can vary between brushings. Moreover, such children are generally not capable of spitting and rinsing properly and therefore ingest a large proportion of the dentifrice used during tooth-brushing [22]. However, these items were added to test whether the mothers were aware of the inability of their young children to spit out paste and rinse their mouths. More mothers answered "no" than "yes" to the item "does the child spit out the dentifrice during brushing?", demonstrating that the mothers are aware of their children's limited brushing skills. However, the item "does the child rinse out his/her mouth during brushing?" revealed that mothers have little knowledge regarding the fact that young children cannot properly perform this act, as nearly all the mothers answered "yes".

There were statistically significant differences between reported habits and observed tooth brushing. A previous study also found differences in the reporting of skin health when evaluated through a telephone interview and mailed questionnaire. When patients were asked whether a non-doctor, such as a partner, had checked his/her skin over their entire body, the prevalence of patients who answered 'yes' for the same question was greater during the telephone interviews than on the mailed questionnaires (p < 0.03) [15]. However, for the determination of food and beverage intake for the assessment of the risk of obesity in young children, parents' reports using a food consumption and physical activity questionnaire demonstrated similar results to a 24-hour dietary log administered by an interviewer (non-significant p-value) [6]. In another study on tooth brushing habits by young children evaluated through interviews, agreement was low between mothers' responses collected at a 6-year interval [9]. In the present study, mothers may have reported better habits than their children customarily have. For instance, the mothers more frequently reported lower quantities on the item "amount of dentifrice dispersed on the brush". However, the observed amount of dentifrice dispersed on the brush was nearly equally distributed. Moreover, 81% of the mothers reported that an adult normally brushed the child's teeth, whereas this figure was only 66% based on the examiner's observations. These findings demonstrate a tendency toward reporting healthier habits.

This difference in the prevalence of habits depending on the method employed may be due to information bias. Some questions may also have confused the mothers as to how they behave and what they believe, which is a problem that can occur in epidemiological surveys [23]. For example, if a mother believes the correct amount of dentifrice is "less than half of the bristles", she may mark this option even if it is not the actual behaviour. Moreover, mothers who have older children may confuse one child's habits with those of another or consider all children's habits equal. Currently, studies are carried out with busy mothers who work and may not be willing to spend much time answering a questionnaire. Moreover, a child may be in the care of grandparents or a baby sitter who may be more familiar with the child's habits.

The observation method should also be evaluated with caution. On the item "who disperses the dentifrice on the brush?", a total of 81.1% of mothers reported that an adult performed this task, whereas the examiner observed this behaviour in 99.5% of the cases (p < 0.001). If one considers that bias is more frequent in reported information, more children would be expected to disperse the dentifrice on the brush themselves when observed by the examiner, which would be closer to reality. However, more mothers did so, indicating that they tended to behave differently in front of the researcher, possibly to appear that they are careful mothers. This item achieved the lowest Kappa agreement (0.00). Considering the formula given in the Methods section, K = po-pe/1-pe [12], the Kappa calculation for this item is expected to be pe = [(38 × 1) + (163 × 200)/201^2 ^= 0.81; K = 0.81-0.81/1-0.81 = 0.0. This explains why this item achieved the lowest Kappa value, while overall agreement was the highest, as overall agreement considers the proportion of agreement by the total (po = 162/201 = 0.81) and not by chance (Kappa).

The present study has limitations that should be considered. There was only a single observed session of tooth brushing. Video recordings of tooth brushing could be an alternative to enable more reliable intra-examiner and inter-examiner comparisons [24]. However, this observation method has been previously used as the gold standard to evaluate another method in a previous study [8].

Although no intra-mother agreement test was performed, the sample was consistent for the investigation of agreement between mothers and observer. The structured form used by the examiner to record tooth brushing habits had exactly the same items and response options in the questionnaire, enabling direct comparison between the two methods. However, this could have accounted for some demonstration effect, where one individual behaves in a certain way after observing and learning the actions of others [25]. Mothers answered the questionnaire one week before performing the observed tooth brushing. Knowing the items addressed in the questionnaire, they could have learned the behaviours that they were expected to perform. The questionnaire may therefore have caused mothers and children to act differently to appear that they are more careful with regard to oral hygiene. More optimistically, mothers may have learned the ideal habits and actually started to behave in a more careful way. Although they were not formally instructed and were left free to perform tooth brushing as they would at home, some unusual behaviour may have occurred.

In the scholastic programme of the day-care centres surveyed, there is only one meeting with parents per semester. It was not possible to schedule another meeting to re-administer the questionnaire, which rendered a re-test assessment impossible. Finally, as the sample comprises young children, some of whom could still be learning or developing the habit (particularly for the items "the child spit the dentifrice out" and "the child rinsed the mouth"), many children may have swallowed the dentifrice because they were unable to spit it out correctly. However, the decision was made to conduct the study with young children because such children are the target population in studies on fluoride intake and the risk of dental fluorosis [4,26,27].

Both methods may have bias and results obtained using them should be interpreted with caution. Although bias is expected in epidemiological studies, excessive bias can invalidate a survey. Therefore, to minimise bias, the data collection process and goals of the study should be explained carefully and exhaustively to the participants, and volunteers should be instructed to be accurate when giving answers. Other strategies could help overcome this problem, such as increased sample size, statistical analysis or the completion of a pilot study prior to the main evaluation. However, neither mothers' reports nor the observation method should be excluded from study designs. Researchers depend on these kinds of data and there is a genuine need for papers addressing reliability. Researchers should be aware of the study limitations and attempt not to influence the participants to adopt biased answers or behaviour. Additionally, the most adequate method should be chosen to fit the goal of the study and must be reliable, valid and cost effective in achieving the intended results, as the wrong choice of method can lead to questionable and conflicting results.

## Conclusion

There was low agreement between observed brushing and mothers' reports. Moreover, the frequency of habits differed depending on whether data were reported or observed, suggesting that data obtained using either of these methods should be considered with caution in epidemiological surveys on fluoridated dentifrice use and the risk of dental fluorosis.

## Competing interests

The authors declare that they have no competing interests.

## Authors' contributions

MJL participated in the study design and data acquisition. CCM participated in the study design, performed the statistical analysis, prepared the first draft and revised the manuscript. SMP and IAP conceived the study, developed the project design and protocols, and revised the manuscript. All authors read and approved the final manuscript.

## Pre-publication history

The pre-publication history for this paper can be accessed here:

http://www.biomedcentral.com/1472-6831/11/22/prepub
